# Supporting Adherence to Antiretroviral Therapy with Mobile Phone Reminders: Results from a Cohort in South India

**DOI:** 10.1371/journal.pone.0040723

**Published:** 2012-08-27

**Authors:** Rashmi Rodrigues, Anita Shet, Jimmy Antony, Kristi Sidney, Karthika Arumugam, Shubha Krishnamurthy, George D'Souza, Ayesha DeCosta

**Affiliations:** 1 Division of Global Health, Karolinska Institutet, Stockholm, Sweden; 2 Department of Community Health, St. John's National Academy of Health Sciences, Bangalore, India; 3 The HIVIND project, St. John's National Academy of Health Sciences, Bangalore, India; 4 Division of Chest Disease, St. John's National Academy of Health Sciences, Bangalore, India; 5 Department of Pediatrics, St. John's National Academy of Health Sciences, Bangalore, India; University of Cape Town, South Africa

## Abstract

**Background:**

Adherence is central to the success of antiretroviral therapy. Supporting adherence has gained importance in HIV care in many national treatment programs. The ubiquity of mobile phones, even in resource-constrained settings, has provided an opportunity to utilize an inexpensive, contextually feasible technology for adherence support in HIV in these settings. We aimed to assess the influence of mobile phone reminders on adherence to antiretroviral therapy in South India. Participant experiences with the intervention were also studied. This is the first report of such an intervention for antiretroviral adherence from India, a country with over 800 million mobile connections.

**Methods:**

*Study design:* Quasi-experimental cohort study involving 150 HIV-infected individuals from Bangalore, India, who were on antiretroviral therapy between April and July 2010. *The intervention:* All participants received two types of adherence reminders on their mobile phones, (i) an automated interactive voice response (IVR) call and (ii) A non-interactive neutral picture short messaging service (SMS), once a week for 6 months. Adherence measured by pill count, was assessed at study recruitment and at months one, three, six, nine and twelve. Participant experiences were assessed at the end of the intervention period.

**Results:**

The mean age of the participants was 38 years, 27% were female and 90% urban. Overall, 3,895 IVRs and 3,073 SMSs were sent to the participants over 6 months. Complete case analysis revealed that the proportion of participants with optimal adherence increased from 85% to 91% patients during the intervention period, an effect that was maintained 6 months after the intervention was discontinued (p = 0.016). Both, IVR calls and SMS reminders were considered non-intrusive and not a threat to privacy. A significantly higher proportion agreed that the IVR was helpful compared to the SMS (p<0.001).

**Conclusion:**

Mobile phone reminders may improve medication adherence in HIV infected individuals in this setting, the effect of which was found to persist for at least 6 months after cessation of the intervention.

## Introduction

Adherence to treatment in HIV is a complex phenomenon influenced by the illness, patient characteristics; healthcare system; treatment regimen and environment [1], . As adherence is critical to treatment success in HIV [5], a number of interventions to support adherence have been implemented. Some of the interventions used to support adherence to antiretroviral therapy (ART) include directly administered ART [6], [7]; financial incentives [8]; education, counseling and social support [9]; electronic and phone reminders [10], [11], [12].

The popularity of mobile phones and their low cost, even in resource constrained settings, has resulted in the use of the technology in healthcare delivery. Examples of evolving uses of this technology in healthcare include data collection, behavior counseling for adherence, disease outbreak tracking and training healthcare workers in remote settings [13], [14]. Of these, recent reports of the use of mobile phones for HIV disease management, including adherence, are available from the Americas and sub Saharan Africa [11], [12], [15], [16], [17].

With 800 million mobile phone connections and the relatively low cost of mobile handsets and services in India [18], mobile communication technology provides a contextually suitable opportunity for antiretroviral adherence support. Approximately 240,000 Indian HIV infected individuals receive first line ART under the National AIDS Control Program [19]. Given the prohibitive costs of second line ART, it is important that the efficacy of first-line regimens is preserved for as long as possible [20] by supporting adherence. We aimed to assess the influence of weekly mobile phone reminders on adherence to antiretroviral therapy in South India and to study their post intervention effects on adherence. Participant experiences with the intervention were also studied. To our knowledge, this is the first such report on mobile phone reminders for adherence support in India.

## Methodology

The study was implemented at the Infectious Disease Clinic, St. John's National Academy of Health Sciences, Bangalore, South India. This is a tertiary level, non profit, private healthcare facility. The clinic provides routine care and treatment to approximately 2,000 HIV infected individuals from within the province of Karnataka and the neighboring provinces of Andhra Pradesh, Tamil Nadu and Kerala. This quasi-experimental cohort study was conducted between March 2010 and July 2011.

The participants included were (i) HIV infected adults who followed up at the clinic as outpatients (ii) had access to a mobile phone and (iii) were on ART for at least a month prior to enrollment. Patients who were participants of other adherence studies were excluded.

First line ART in the setting of India's National AIDS Control Program consists of zidovudine or stavudine, plus lamivudine plus nevirapine (or efavirenz in patients on anti tuberculosis treatment) to be taken by the patient twice daily [21]. These antiretrovirals are available free of cost to HIV infected individuals through a network of public healthcare facilities and public private partnerships. Our intervention was restricted to individuals on such first line regimens.

The intervention studied was adherence support with mobile phone reminders. Each reminder comprised of two components, (i) an interactive voice response (IVR) call and (ii) a non interactive neutral picture delivered as a short message service (SMS). All participants received both components of the intervention once a week for 6 months from the date of enrollment. Each component was received on two separate days in a week at a time chosen by the participant. Both components were demonstrated to participants at recruitment. All participants were trained to respond to the IVR and to access the pictorial message.

The interactive call component required participants to respond to the question “have you taken all your medicines yesterday?” with a “1” if they had not missed any doses in the previous 24 hours and “2” if they had. If participants missed the call, three additional calls were made over the ensuing 24 hours, providing participants with an opportunity to receive and respond to the call. However, in this study, patient responses were not used as measures of adherence but were used only to make the reminder interactive. Online, web-based interfaces captured for each participant, (i) the status of IVR delivery and receipt (ii) the participant responses to the IVR. For the IVR, participants could choose one of five languages i.e.; English, Kannada, Telugu, Tamil or Hindi. These languages were those commonly spoken in south India.

The SMS was a line diagram of a lamp. There was no text included in the SMS. All participants received the same picture SMS throughout the intervention period. The SMS delivery status was captured by the online web based interface for each participant.

### Instruments and follow up

Demographic details and prior experience with mobile phones were ascertained at enrollment. The outcome measure studied was change in adherence over a 12 month period (6 months during the intervention and 6 months after discontinuation of the intervention). Adherence at baseline, during and after the intervention was assessed using the pill count. Pill count was measured at baseline, followed by month 1, month 3, month 6 during the intervention, and at two time points post intervention i.e. month 9 and month 12. At each time point, adherence for the previous month was measured. Adequate adherence was defined a priori as having consumed ≥95% of ART pills for that month. Pill count was then calculated as a proportion; i.e. the number of pills taken over the last month divided by number of pills expected to be taken as per prescription and expressed as a percentage. Barriers to adherence such as reasons for missing medication doses were also recorded at baseline and at each follow up visit during the intervention period using the ‘ACTG barriers to adherence self report (ACTG ql0777)’ [22]. Both the pill count and barriers to adherence were assessed by trained research assistants not involved in routine care of the patient. Participant experiences with the intervention in terms of receipt, usefulness, privacy and intrusion were assessed on a five-point Likert scale at the end of the intervention period.

### Statistical analysis

Demographic characteristics, adherence and participant experiences were expressed using frequencies; measures of central tendency and dispersion. Binary logistic regression was used to assess the association between socio-demographic variables and baseline adherence.

Adherence, the main outcome variable, had some missing data (described below) due to participants lost to follow-up. We therefore used three approaches to assess the influence of the intervention on adherence. These approaches to data analysis were (i) complete case analysis, where, cases with missing outcome measures were excluded from the analysis (ii) missing equals adequately adherent, i.e. all cases with missing adherence measures at any time point were considered as “adequately adherent” and included in the analysis and (iii) missing equals not adequately adherent, i.e. all cases with missing adherence measures at any time point were considered “not adequately adherent” at that time point and included in the analysis. Cochran's Q for *k* related samples and Mc Nemar's test with Bonferroni correction were used to study change in adherence over time. The level of significance for change in adherence was defined as p<0.003. The Wilcoxon signed rank test was used to compare participant experiences at 6 months with respect to the IVR and SMS. Ordinal regression was used to compare participant experiences with socio-demographic variables.

The study was approved by the institutional review board at St. John's National Academy of Health Sciences, Bangalore, India. Written informed consent was obtained from all participants prior to enrollment.

## Results

Of the 231 eligible participants invited to participate in the study, 150 enrolled. Reasons for non participation included time constraints (33%) followed by disinterest (25%) and stigma (13%). **N**on participants were similar to participants with respect to age, sex, marital status, education, time since diagnosis, duration on ART, type of regimen, and baseline CD4 counts. However, a larger proportion of nonparticipants (31%) were rural residents in comparison to the participants (10%).

Retention rate in the study at month 12 was 94%. The participant characteristics at baseline are described in Table 1. Two thirds of the participants were men. This reflected the sex distribution of patients attending the clinic at the time of enrollment. Table 1 also describes the associations between studied participant characteristics and baseline adherence.

**Table 1:** Demographic profile and association with baseline adherence.

Characteristics	Frequency	≥95% adherence at baseline	Association with baseline adherence	
	N = 150		AOR^d^	p value
**Sex**				
Male sex	109(73%)	96(88%)	2.404	0.327
Female	41(27%)	33(80%)	Referent	
**Residence**				
Urban	135(90%)	116(86%)	1.823	0.535
Rural	15(10%)	13(87%)	Referent	
**Education**				
Formal	142(95%)	122(86%)	0.173	0.172
No formal Education	8(5%)	7(88%)	Referent	
**Currently employed**				
Yes	123(82%)	109(87%)	2.806	0.194
No	27(18%)	20(74%)	Referent	
**Age(mean ± SD) (years)**	38.54**±**7.7		0.982	0.658
**Median Income** (IQ range) (USD)^a^	10(60–200)		1.000	0.786
**Martial Status**				
Married	109(73%)	93(85%)	0.476	0.306
Others^b^	41(27%)	36(88%)	Referent	
**Literacy**				
Literate	141(94%)	123(87%)	9.361	0.027
Cannot read and write in any language	9(6%)	6(67%)	Referent	
**Use of phone functionality**				
Knew how to make Calls	149(99%)			
Knew how to receive SMS				
Yes	127(85%)	110(87%)	1.676	0.516
No	23(15%)	19(83%)	Referent	
Knew how to send SMS				
Yes	71(47%)	61(86%)	0.710	0.577
No	79(63%)	68(86%)	Referent	
**Clinical profile (HIV)**				
Median CD4 cells/mm^3^ (IQ range)	437 (255–644)		0.999	0.634
Median months since diagnosis (IQ range)	28(13–65)		1.017	0.104
***HIV Stage***				
Stage 1 and 2	111(79%)	95(87%)	Referent	
Stage 3 and 4	39(21%)	34(87%)	1.229	0.761
**Antiretroviral Therapy**				
Regimen 1(ZDV/d4T+3TC+NVP)^c^	136(91%)	117(86%)	1.399	0.752
Regimen 2 (ZDV/d4T+3TC+EFV)^c^	14(9%)	11(79%)	Referent	
Median months on ART (IQ range)	14 (18–27.75)		1.022	0.395
**Cotrimoxazole prophylaxis**				
Yes	75(50%)	66(88%)	1.335	0.634
No	73(49%)	61(84%)	Referent	
**On Anti-tuberculosis treatment**				
Yes	6 (5%)	5(83%)	1.258	0.897
No	144(95%)	127(88%)	Referent	
**Mean weight (Kg)**	59.45±12.66		1.008	0.714

a: USD = US Dollar, 1USD ≈ 50 Indian Rupees,

b = Single, divorced, separated,

c: ZDV = Zidovudine, d4T = Stavudine, 3TC = Lamivudine, NVP = Nevirapine, EFV = Effavirenz,

d: AOR = Adjusted odds ratio,

A total of 4,103 calls were expected to be delivered to the participants during the 6 months of the intervention. Of these 204 (5%) of the calls were not delivered due to technical problems with the service; thus 3,895 calls were made to the participants. Of these, 3372 (87%) calls were received by the participants. Of the 3372 calls received by the participants, the “1” (taken all pills) response was recorded for 2725 (81%) calls, the “2” (not taken pills) response was recorded for 43 (1%) calls while the rest of the calls (18%) recorded an erroneous response (any other number).

A total of 3,073 picture messages were delivered to the participants during the study. For month 6 of the intervention, 89 (59%) participants reported that they had viewed all the messages, while 20 (13%) persons reported that they had viewed none of the messages in the last month of the intervention. Of the 20 participants who did not view their messages, 16 made limited use of the SMS feature, and 11 reported that they did not know how to use the SMS feature on their mobile phones despite being trained at enrollment.

Nine (6%) of the 150 participants had missing adherence data at different time points. Of these nine participants, three were transferred out to another clinic for second line ART, four were transferred to clinics closer to their homes for logistic reasons and two could not be traced.

**Figure 1:**
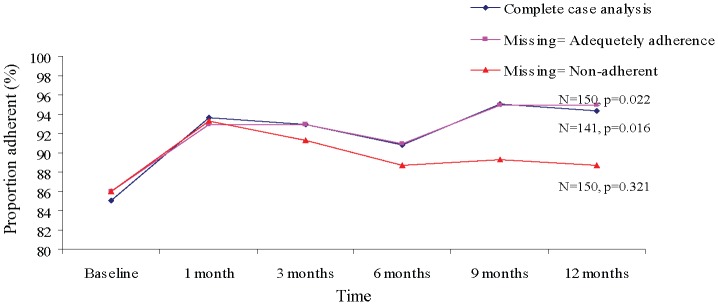
Proportion of study participants adherent over time. The figure shows the change in proportion of participants adherent over time during the study period (1 year) based on the 3 analytical approaches used in the i.e. complete case analysis, missing equals adherent and missing equals non adherent.

Complete case analysis for 141 participants with complete data showed that those adequately adherent at baseline, month 1, month 3, month 6, month 9 and month 12 were 120 (85%), 132 (94%), 131 (93%), 128 (91%), 134 (95%), and 133 (94%) respectively. There was a significant improvement in proportions adherent over time (p = 0.016) (Fig. 1).

Using the second approach where missing adherence data at any time point was considered “adequately adherent” at that time point, participants adherent were 129 (86%) at baseline, 140 (93%) at month 1, 139 (93%) at month 3, 136 (91%) at month 6, 143 (95%) at month 9 and 144 (96%) at month 12. A significant increase in proportions adequately adherent over time was also seen with this approach (p = 0.022).

The third approach, where missing adherence data at any time point were considered as “not adequately adherent”, showed that, participants adequately adherent were 129 (86%) at baseline, 140 (93%) at month 1, 137 (91%) at month 3, 133 (89%) at month 6, 134 (89%) at month 9, and 133 (89%) at month 12. No significant increase in proportions adequately adherent over time was seen with this approach (p = 0.321) (Fig. 1).

### Barriers to adherence

Forgetfulness was the most common reason for non-adherence throughout the study. Seventeen percent of the participants reported forgetfulness as a barrier at enrollment. However, this significantly decreased from baseline with time during the intervention period (17%; 10%; 6% and 3% at baseline, 1 month, 3 months, and 6 months respectively, p<0.001).

### Experiences with the intervention

Participant experiences with the two components of the intervention are described in Table 2. Of 136 participants who reported their experiences with the intervention at the end of month 6, 23 reported not receiving all their calls in the last 4 weeks. Of these 23, 12 reported technical issues with the mobile phone/handset and 11 reported being away from their phone. All 136 participants considered the IVR easy to use. A significantly higher proportion of participants agreed that the IVR was helpful compared to the SMS (p<0.001). Both the SMS and the IVR were considered comparable in terms of privacy and intrusion (Table 2). Ordinal regression showed no association between participant experiences and demographic variables listed in Table 1. *Preference:* Of the 136 respondents, 42 (34%), preferred only the phone call, 15 (11%) preferred only the SMS, 60 (44%) preferred both the phone call and the SMS, while the rest preferred neither.

**Table 2:** Experiences with the intervention at 6 months follow-up: IVR Vs SMS (n = 136).

Statement	Component	Strongly Disagree 1	Disagree 2	Neutral 3	Agree 4	Strongly agree 5	p value*
Intervention helps take medicines	IVR	0	4 (3%)	12 (9%)	120 (88%)	0	
	SMS		4 (3%)	20 (15%)	111 (82%)	1 (1%)	<0.001
Work hard to ensure intervention is not accessed	IVR	0	102 (75%)	20 (15%)	12 (9%)	2 (1%)	
	SMS	105 (77%)	14 (10%)	16 (12%)	1 (1%)	0	0.183
Feel ashamed if intervention was accessed by others	IVR	1 (1%)	104 (76%)	10 (7%)	20 (15%)	1(1%)	
	SMS	110 (81%)	11 (8%)	14 (10%)	1(1%)	0	0.115
Intervention is an intrusion	IVR	1 (1%)	123 (90%)	4 (3%)	8 (6%)	0	
	SMS	1 (1%)	120 (88%)	9 (7%)	6 (4%)	0	0.816

* p value by Wilcoxon signed rank test.

## Discussion

Based on the analytical approach used the effect of the intervention on adherence (change in adherence) showed differing results. A complete case analysis showed beneficial effect of the intervention on adherence. Similar results were obtained when subjects with missing data were considered adequately adherent. On the other hand, when subjects with missing data for adherence were considered non-adherent, no significant influence of the intervention was observed. While it is reported that patients who miss clinic attendance for medication refills were more likely to be non-adherent [23], this may not necessarily be the case in our study as seven of nine patients with missing values were transferred to other treatment centers for either second line ART or logistic reasons (i.e.; proximity to their homes). These seven patients may or may not have been adherent to treatment after transfer. On the contrary, two of the nine participants who did not complete the study could not be traced, and hence, were considered completely lost to follow-up (LFU). It is more likely that these two participants were not adequately adherent to treatment [23]. The beneficial effect of the intervention on adherence was also observed, on excluding from the analysis, the seven participants who were transferred to other treatment centers (p<0.029 when LFU = not adequately adherent and p<0.011 when LFU = adequate adherence).

Though there are reports of improvement in adherence with interventions for supporting adherence, only a few studies have addressed their post intervention effect [24], [25]. A study done on adolescents in Los Angeles, USA, reported a decline in adherence and increase in viral load in HIV infected adolescents when daily cell phone reminder calls were tapered over a period of 24 weeks [26]. The participants in this study were eight youth aged 16–24 yrs most of who reported substance abuse. These participant characteristics possibly influenced the decline in adherence while tapering the intervention. Our results differed from the Los Angeles study possibly due to different participant demographics and study contexts. A randomized controlled trial (RCT) from Pakistan showed improvements in adherence for up to 2 weeks after the cessation of weekly phone calls for a month [27]. The results were similar to those in our cohort, though the post intervention effect was assessed earlier. However, participant demographics like age structure and sex distribution were similar to those in our study. In another study from the United Kingdom, the sustained effect of pagers used to improve memory, seven weeks after their withdrawal, was attributed to establishment of routines [27]. Participants in this study had problems with memory, attention, or organizational issues. Electronic devices like pagers are thought to provide consistent and reliable prompts that may be difficult for family members to provide. We did not assess memory and its effect on adherence in our study. However the weekly reminders probably enabled establishment of routines, especially in patients more recently initiated on ART.

As in our study, individual sociodemographic factors were not found to be associated with adherence in another setting in Bangalore, India [28]. Forgetfulness was the most common cause of non-adherence in our study and has been reported in literature [2], [29], [30]. A study from California, USA, recommends the use of programmable electronic medication reminders that increase the quality, distinctiveness and relatedness of external cues to minimize forgetfulness and improve adherence in HIV [31]. Further evidence to support this theory is available from reports of improved adherence with the use pagers [32], watch alarms [33], phone calls [26], and SMS reminders [11] to support adherence [34]. A study on tuberculosis, HIV and syphilis from North Carolina reported a significant correlation between forgetting one's medications and considering SMS medication reminders helpful [35]. Reports of forgetfulness reduced with time during the intervention in our study. On the contrary digital reminders for improving adherence to ART were not found effective in an RCT in Nairobi, Kenya. This study reported intensive early adherence counseling to have a sustained effect on adherence in comparison to digital reminders [36]. The interactiveness of the intervention in our study was probably the reason for the difference in effect observed between the two studies.

Recent studies of mobile phones for adherence support in HIV have used SMS technology. In an RCT in Kenya, HIV infected research participants were expected to call their healthcare worker in response to an SMS query regarding their health [11]. Another study from Kenya independently reported a significantly larger proportion of adherent participants exposed to SMS reminders in comparison to controls [12]. A study from Boston, USA, also reported better adherence to ART in participants receiving personalized SMS reminders in comparison to controls with beepers [17]. Most of these studies used SMS reminders unlike our study that used a combination of IVR and SMS reminders. We combined IVR and SMS reminders as a pilot study in our setting showed that patients preferred voice calls over SMSs [37]. We confirmed this preference for IVR technology by patients one month after initiation of the intervention [38]. RCT of IVR and SMS based adherence support interventions are underway in Cameroon and India, the results of which are awaited [39], [40].

Call completion rates of 75–87% have been reported by studies that have used IVR technology for adherence support, and were similar to the call completion rates in our study [17], [41]. As previously reported, our study also identified technical challenges with the phones and being away from their phones as reasons for not answering the calls [12].

The IVR technology in our study was considered easy to use. Previous studies involving IVR technology with voice recognition have reported difficulties in decoding participant responses. This resulted in persistence of the call and frustration on behalf of the participant [42]. Additionally, participants have found both IVR and SMS prompts difficult to follow [42]. The simplicity of the intervention used in our study probably made it easy to use.

We also studied intrusiveness and privacy concerns with the two components of the intervention. Weekly, IVRs and neutral SMSs timed to the participant's convenience probably reduced intrusion and enhanced the privacy of the intervention. Unlike our study, the study from Los Angeles reported that HIV patients found cell phone reminder calls to be intrusive [43]. Both, the IVR and SMS were comparable in terms of intrusion and privacy in our study, despite concerns in literature to the contrary [44]. Even though comparable, IVR calls were preferred to SMSs and considered more helpful by participants. This is probably due to the generally lower use of the SMS function by participants in our study. Similar findings, reported by previous studies in the same setting, were the basis of our intervention [37], [40]. A study from Uganda reported illiteracy, language barriers and possibility for direct communication as reasons for preferring voice calls to SMSs in other resource limited settings [45]. The ability to choose the language and the interaction of the IVR probably enhanced its preference in our study. In contrast, unacceptability of IVR calls due to an inability to speak to a real person was reported from Quebec, Canada [42]. The role of personal interaction in adherence improvement has been confirmed by a study from Cape Town, South Africa [46].

Although adherence was the outcome of interest in this study, the technology also has the potential to improve other aspects of HIV care like clinic attendance [23]. It can also be explored for improving adherence in communicable and chronic disease like tuberculosis and diabetes [35], [47].

### Methodological issues

(i) The clinic setting, close follow-ups and routine ART counseling, may have resulted in the relatively high adherence rates at baseline. Despite the clinic setting and high adherence rates at baseline, we observed improvements in adherence during the study. This implies that the benefits of the intervention could possibly be greater in general clinic settings where adherence rates may be lower [48]. (ii) Also participants could have been more motivated than non participants to maintain adequate adherence. However, background characteristics of non participants were similar to participants in our study, except that the former were more rural. (iii) Adherence is affected by duration on treatment [49]. In our study the duration on ART varied from one to 86 months, this could have affected their adherence ART. (iv) As patients were under study and so closely followed up, the Hawthorne effect on adherence cannot be ruled out [50]. An RCT of the intervention will provide stronger results and enable assessment of clinical and virological outcomes that were not assessed in our study. (v) The different approaches to analysis of change in adherence in our study provided differing results [51]. We have alluded to this at the beginning of the discussion.

Overall, our **s**tudy contributes to the growing body of evidence on the capacity of mobile phone reminders to influence medication adherence in HIV infected individuals. While the findings of our study vary depending upon the analytical approach used, a complete case analysis indicates significant effect of the intervention on adherence. Despite the limitations of this study as discussed, given the ubiquity of mobile phones in the Indian setting and the simplicity of the intervention, the use of mobile phones for support of adherence to antiretroviral therapy holds promise and is worthy of further exploration in the Indian context.
